# A rare occurrence of osteoblastoma in a child

**DOI:** 10.4103/1817-1745.76118

**Published:** 2010

**Authors:** Pavan Kumar Avadhanam, Sreedhar Vuyyur, Manas Kumar Panigrahi

**Affiliations:** Department of Neurosurgery, Nizam’s Institute of Medical Sciences, Hyderabad, India

**Keywords:** Benign tumors of spine, osteoblastoma, osteoblastoma in childhood

## Abstract

To report a rare occurrence of osteoblastoma involving the L4 vertebra in an 8-year-old female child with histological features suggestive of osteoblastoma with secondary aneurysmal changes. The mean age incidence of osteoblastoma is 20.4 years. In our case, a rare presentation of osteoblastoma was seen in the first decade. The child was admitted with a 1-year history of increasing back pain and radiculopathy. The child was evaluated with X-rays, computed tomography scan and magnetic resonance imaging, which indicated involvement of the posterior elements of the 4^th^ lumbar vertebrae. Decompression of the L5 nerve and resection of the tumor was performed. Osteoblastoma is a rare tumor with an incidence of 1% of all tumors and 30–40% of cases involving the spine. Osteoblastoma occurs most commonly in males (M:F, 2.5:1). The most common area of involvement is the cervical spine followed by the lumbar spine. Posterior elements of the vertebrae are commonly involved.

## Introduction

Osteoblastoma is a benign vascular bone-forming tumor. It was independently described by Lichtenstein[1] and Jaffe.[2] Imageologically, these are osteolytic lesions larger than 2 cm with little or no evidence of perifocal sclerosis. Osteoblastomas account for approximately 1% of all primary bone tumors. About 30–40% of all cases of osteoblastoma involve the spine. The most common area of involvement is the cervical spine (20–40%) followed by lumbar spine. In the spine, most often the osteoblastoma is confined to the posterior elements. The mean age of presentation is 20.4 years, with case reports from 5 to 72 years. The main clinical feature is pain, followed by neurological symptoms and scoliosis. Frequently, there is an invasion of the epidural space surrounding the nerve roots and the cord leading to radiculopathy or cord compression. Recurrence rates after resection are described up to 10%.[3–7] Some authors have reported the possibility of malignant transformation.[8–10] Surgery is aimed at complete resection and protection of the sensitive neuroanatomic structures.

## Case Report

An 8 year old female child came to us in nov 2008 with complaints of back pain and restriction of lower back movements since 1 year. Initially, the pain was aggravated on standing and while walking. Later, she had rest pain. She also had tingling and numbness along the dorsal aspect of her left leg since 6 months. This sensory disturbance was associated with slippage of footwear while walking.

On examination, there was localized swelling in the L4 region on the left side. There was also paraspinal muscle spasm. Lumbar spine movements were restricted. Localized and diffuse tenderness was present. Muscle power in extensor hallucis longus and extensor digitorum longus was grade 4 out of 5 in the left lower limb. Superficial sensory functions in the left L5 dermatome were diminished. Muscle stretch reflex in the left ankle was sluggish.

### Radiological Investigations

**Figure 1:**
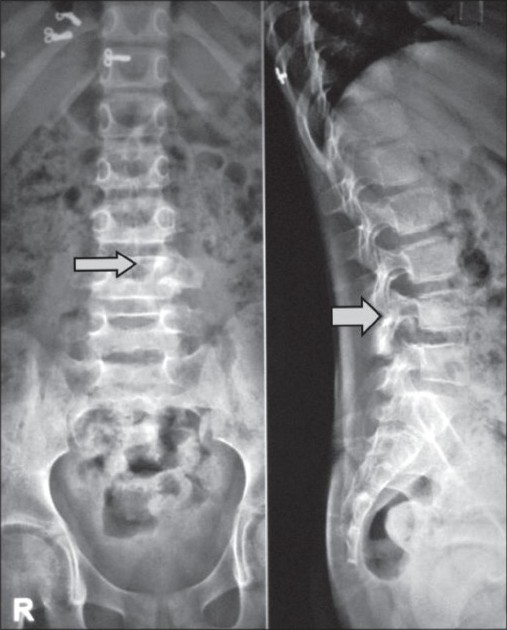
Plain X-ray showing expansion of the left fourth lumbar pedicle: transverse process and superior facet

**Figure 2:**
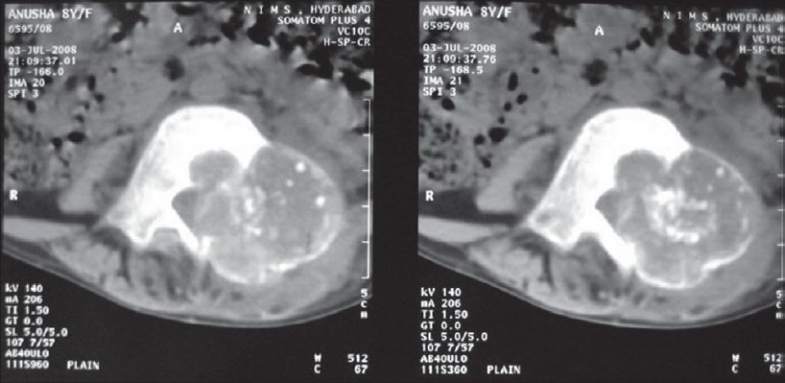
Computed tomography scan images shows expansive osteolytic lesion with thin rim of cortex involving the spinous process, lamina, pedicle, transverse process and even into the body. Calcification is well defined

**Figure 3:**
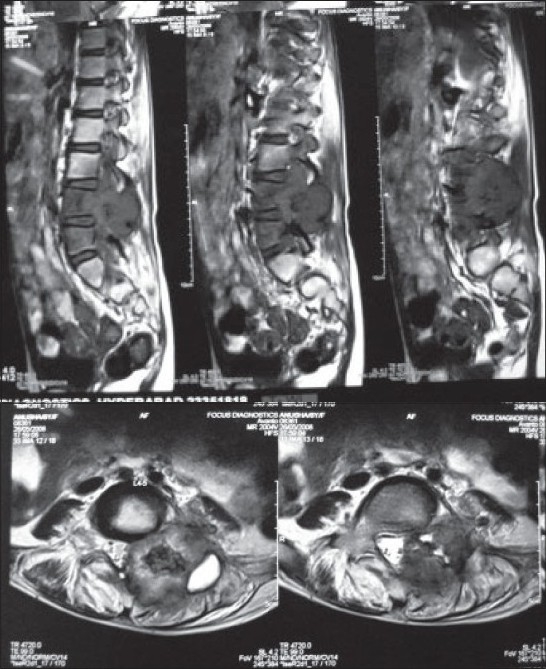
Magnetic resonance imaging images show the lesion in posterior elements with edema extending into the spinal canal, L4, L5 vertebral bodies and intervening disc

We thoroughly investigated the patient with plain roentgenograms, computed tomography (CT) scan and magnetic resonance imaging (MRI). Plain roentgenogram showed expansion of the left fourth lumbar pedicle, superior articular facet and transverse process[Figure 1]. Flecks of calcification were noted. Expansive osteolytic lesion with thin rim of cortex was found in the CT scan involving the spinous process, lamina, pedicle, superior articular process, transverse process and even into the bodyp[Figure 2]. Calcification was well defined in the CT scan. MRI images demonstrated lesion in the posterior elements with edema extending into the spinal canal, L4, L5 vertebral bodies and intervening disc[Figure 3]. Compression of thecal sac and L5 nerve root were seen. According to the Enneking score of benign musculoskeletal lesions, our tumor was classified as grade 2.

### Intraoperative Features

We found a firm, reddish to brown mass extending from the spinous process to the pedicle. Pseudocapsule with little vascularity was seen surrounding the mass. The tumor mass was friable. Complete excision of the tumor was possible with pseudocapsule in toto. There was no evidence of any necrosis or cystic spaces in the tumor.

### Postoperative Course

The patient was relieved of back pain. The patient was eased of signs of neurological compression of the 5^th^ lumbar nerve. No fresh deficits were noted postoperatively.

### Histopathological Report

#### Gross description

Multiple grey white to grey brown soft tissue bits with bony spicules.

#### Microscopic description

**Figure 4:**
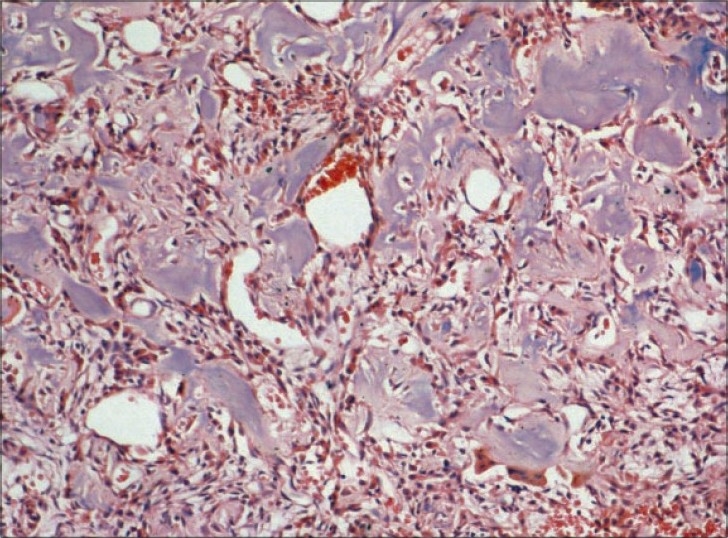
Histological sections showed a lesion comprised of haphazardly arranged woven bone trabeculae within a richly vascular stroma (H and E, ×100)

**Figure 5:**
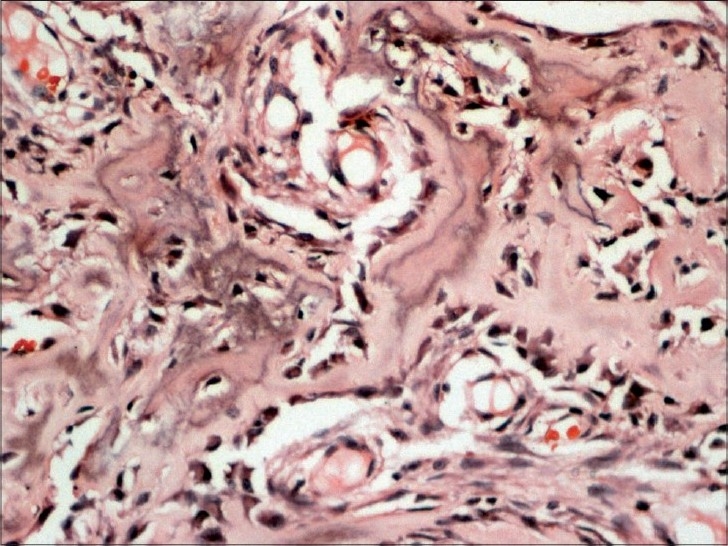
Higher magnification showing bony trabeculae with osteoblastic rimming (H and E, ×200)

**Figure 6:**
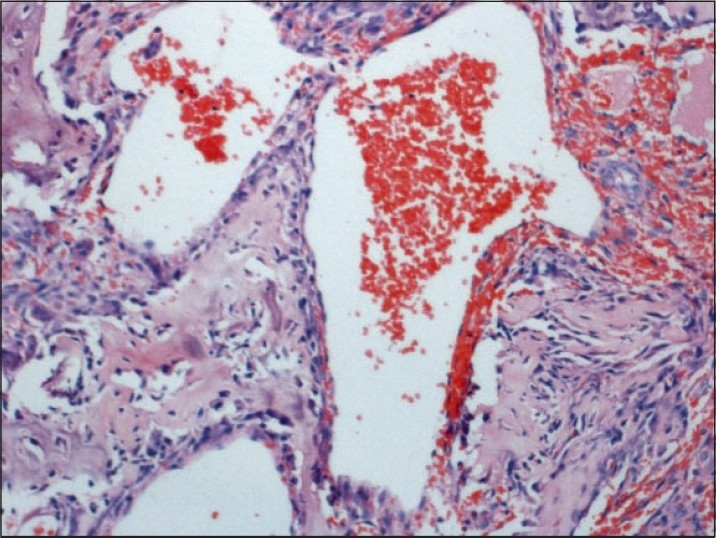
Areas of secondary aneurysmal cyst change represented by dilated cystic spaces filled with red blood cells (H and E, ×100)

Multiple sections show irregular interconnecting trabeculae of woven bone within a fibrous stroma[Figure 4]. The trabeculae show prominent osteoblastic rimming and, at places, the osteoblasts show an epitheloid appearance[Figure 5]. Stroma consists of spindle cells, thin-walled capillaries and fibrous tissue. Focally, stromal spindle cells show a storiform pattern. Multinucleated osteoclast giant cells are also seen. In addition, there are foci showing an aneurismal bone cyst (ABC) change and osteoclast giant cells[Figure 6]. There is no significant pleomorphism and mitotic activity in any of the cellular components. The features are consistent with “osteoblastoma with secondary ABC changes.”

## Discussion

Osteoblastoma is a rare bone tumor and accounts for approximately 0.5–1% of all primary bone tumors.[11–13] Spinal involvement is described in 30–40% of all cases.[11,13–15] In 20–40% of those, the cervical spine is affected.[3,13,15,16] Lumbar spine is the next most common affected area. Osteoblastoma is most frequently found in the posterior elements.[15,17–19] Pain is usually the main clinical symptom, followed by neurologic symptoms, scoliosis and torticollis.[13,15,16] Compared to osteoid osteoma, there is a higher rate of neurological deficits.[4,15,16,20] The large tumor mass can be associated with compression of the vertebral arteries if the tumor arises in the cervical spine. Osteoblastoma should be ruled out if patients present with neurological symptoms and pain over extended periods of time, especially at night.

Delay of diagnosis is very common, and seems to occur on average 6–12 months or later following the initial presentation.[13,16,21–23] The treatment of choice for osteoblastoma is complete surgical resection. Preoperative interdisciplinary cooperation is necessary among the radiologist, neurosurgeons, vascular surgeons and orthopedic surgeons. Recurrence rates are described up to 10%, especially in Enneking Grade 3 lesions.[3,4] In some cases, there is a possibility of malignant transformation.[8–10] If complete resection is not possible, radiotherapy and, in some cases, chemotherapy seem to be alternative treatment options.[24–29]
